# CD38 inhibition by apigenin ameliorates mitochondrial oxidative stress through restoration of the intracellular NAD^+^/NADH ratio and Sirt3 activity in renal tubular cells in diabetic rats

**DOI:** 10.18632/aging.103410

**Published:** 2020-06-07

**Authors:** Yoshio Ogura, Munehiro Kitada, Jing Xu, Itaru Monno, Daisuke Koya

**Affiliations:** 1 Department of Diabetology and Endocrinology, Kanazawa Medical University, Ishikawa, Japan; 2 Division of Anticipatory Molecular Food Science and Technology, Medical Research Institute, Kanazawa Medical University, Uchinada, Ishikawa, Japan; 3 Department of Endocrinology and Metabolism, The Affiliated Hospital of Guizhou Medical University, Guiyang, Guizhou, China

**Keywords:** diabetic kidney disease, CD38, Sirt3, mitochondrial oxidative stress

## Abstract

Mitochondrial oxidative stress is a significant contributor to the pathogenesis of diabetic kidney disease (DKD). We previously showed that mitochondrial oxidative stress in the kidneys of Zucker diabetic fatty rats is associated with a decreased intracellular NAD^+^/NADH ratio and NAD^+^-dependent deacetylase Sirt3 activity, and increased expression of the NAD^+^-degrading enzyme CD38. In this study, we used a CD38 inhibitor, apigenin, to investigate the role of CD38 in DKD. Apigenin significantly reduced renal injuries, including tubulointerstitial fibrosis, tubular cell damage, and pro-inflammatory gene expression in diabetic rats. In addition, apigenin down-regulated CD38 expression, and increased the intracellular NAD^+^/NADH ratio and Sirt3-mediated mitochondrial antioxidative enzyme activity in the kidneys of diabetic rats. *In vitro*, inhibition of CD38 activity by apigenin or CD38 knockdown increased the NAD^+^/NADH ratio and Sirt3 activity in renal proximal tubular HK-2 cells cultured under high-glucose conditions. Together, these results demonstrate that by inhibiting the Sirt3 activity and increasing mitochondrial oxidative stress in renal tubular cells, CD38 plays a crucial role in the pathogenesis of DKD.

## INTRODUCTION

Diabetic kidney disease (DKD) is a serious diabetic microvascular complication and the leading cause of end-stage kidney disease (ESKD). Since in type 2 diabetic patients, the renal damage is induced by multiple metabolic risk factors, including hyperglycemia, hypertension, dyslipidemia, and over-nutrition/obesity, multifactorial management of all metabolic risk factors is recommended [1–3]. However, even when patients undergo the multifactorial management, the therapy is often insufficient to suppress the progression of DKD, and there is still a residual risk of progression to ESKD.

Renal tubular damage is closely associated with the pathogenesis of DKD, and is recognized as a “diabetic tubulopathy” [4, 5]. Since a large number of mitochondria reside in renal tubular cells to meet the high energy demand necessary for the reabsorption of nutrients, they are an important source of reactive oxygen species (ROS) in the kidney [6]. In the diabetic state, the mitochondrial function in tubular cells may be disrupted by increased energy demand due to the excessive reabsorption of glucose and sodium [7]. Therefore, protecting tubular cells against mitochondrial oxidative stress in diabetic kidneys might serve as a therapeutic strategy to preserve the renal function. Mitochondrial oxidative stress occurs due to the imbalance between ROS production and anti-oxidative capacity [8]. We have previously reported that mitochondrial oxidative stress is induced by decreased superoxide dismutase 2 (SOD2) and isocitrate dehydrogenase 2 (IDH2) activities associated with a reduced intracellular NAD^+^/NADH ratio and Sirt3 activity in the kidneys of type 2 diabetic rats [9]. Moreover, the reduced intracellular NAD^+^/NADH ratio and Sirt3 activity were accompanied by an increased renal expression of the NAD^+^degrading enzyme CD38 [10, 11]. Previous reports have shown that CD38 knockout mice have higher NAD^+^ levels, and are protected against high fat diet-induced obesity and metabolic syndrome [12]. Activity of CD38 increases during aging, and this is associated with age-related decline in NAD^+^, reduction in Sirt3 activity, and mitochondrial dysfunction in liver, adipose tissues, and skeletal muscles [13]. However, it remains unclear whether the increased expression of CD38 is involved in the pathogenesis of DKD caused by mitochondrial oxidative stress.

Apigenin (4,5,7-trihydroxyflavone) is a flavonoid present in vegetables (parsley, celery, and onions), fruits (oranges), herbs (chamomile, thyme, oregano, and basil), and plant-based beverages (tea, beer, and wine) [14–16]. A previous study has shown that apigenin inhibits CD38, thus increasing NAD^+^ levels, and improving glucose and lipid homeostasis in obese mice [17]. However, there have been few reports evaluating the effect of apigenin on DKD.

Here, we show for the first time that CD38 plays a crucial role in mitochondrial oxidative stress by reducing the NAD^+^/NADH ratio and Sirt3 activity in the kidneys of type 2 diabetic rats. The NAD^+^/NADH ratio and mitochondrial anti-oxidative properties mediated by Sirt3 activation are restored by apigenin, leading to the amelioration of diabetes-induced renal injury, particularly renal tubular injury. We believe that these findings may lead to a novel strategy for the treatment of diseases characterized by an imbalance in NAD^+^ metabolism, including DKD.

## RESULTS

### Characteristics of the experimental rats

To evaluate the role of CD38 in DKD, male Zucker Diabetic Fatty Rats (ZDFRs) and male Zucker Lean Rats (ZLRs) were treated with the CD38 inhibitor apigenin, or control saline solution. The characteristics of the rats at the end of the experiment are shown in Table 1. There was no significant change in whole body weight among the four groups of rats. The ZDFRs that received saline exhibited significantly elevated levels of HbA1c and increased kidney weight compared to the ZLRs that received saline. Treatment with apigenin reduced the HbA1c values, but did not change the kidney weight in the ZDFRs. The serum levels of cystatin C were not significantly changed among the groups. The ratios of urinary albumin/creatinine (Cr), liver-type fatty acid-binding protein (L-FABP)/Cr, and 8-hydroxy-2’-deoxyguanosine (8-OHdG)/Cr were significantly higher in ZDFRs treated with saline compared to ZLRs treated with saline. The ZDFRs treated with apigenin exhibited significantly reduced ratios of urinary albumin/Cr, L-FABP/Cr, and 8-OHdG/Cr compared with the ZDFRs that received saline. There were no significant changes in whole body weight, kidney weight, HbA1c values, serum cystatin C levels, ratios of urinary albumin/Cr, L-FABP/Cr, and 8-OHdG/Cr between the ZLRs that received saline and the ZLRs treated with apigenin.

**Table 1:** Characteristics of the rats at the end of the experiment.

	**ZLRs (n=6)**	**ZLRs + Apigenin (n=4)**	**ZDFRs (n=6)**	**ZDFRs + Apigenin (n=6)**
Body weight (g)	393.8±15.6	379.0±12.0	398.5±49.1	392.3±26.0
HbA1c (%)	3.47±0.05	3.42±0.09	10.4±0.18 *	9.47±0.57
Kidney weight (g)	2.53±0.14	2.57±0.12	3.56±0.25 ^#^	3.38±0.18
U-Alb/Cr (mg/gCr)	0.018±0.001	0.010±0.003	1.52±1.00 *	0.34±0.08
U-L-FABP/Cr (ug/gCr)	0.47±0.04	0.98±0.85	29.1±1.40 *	7.61±4.40
Cystatin C (ng/mL)	0.92±0.04	1.13±0.06	1.14±0.07	1.04±0.12
U-8-OHdG/Cr (ng/mgCr)	9.30±1.33	7.30±2.00	43.6±20.9 *	13.5±4.82

*p<0.01 vs. other groups. ^#^p<0.01 vs. ZLRs and ZLRs + Apigenin.

ZLRs; Zucker lean rats, ZDFRs; Zucker diabetic fatty rats; U-Alb/Cr; urinary albumin/creatinine ratio, U-L-FABP; U-L-FABP/Cr; urinary liver-type free fatty acid binding protein/creatinine ratio, U-8-OHdG/Cr; urinary 8-hydroxy-2’-deoxyguanosine/creatinine ratio.

### Apigenin ameliorates renal fibrosis and pro-inflammatory gene expression in diabetic rats

**Figure 1:**
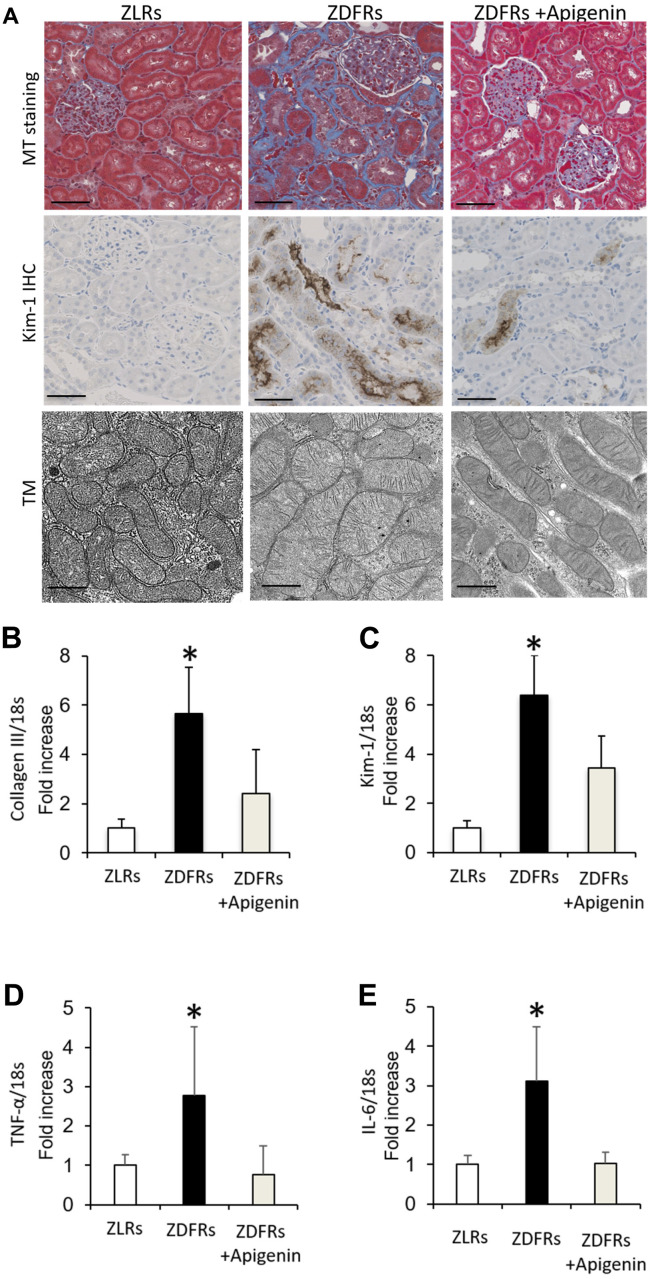
**Apigenin ameliorates renal fibrosis and inflammatory gene expression in diabetic rats.** (**A**) Representative photographs of MT staining (scale bar: 100 μm) and IHC of Kim-1 in the tubulointerstitial area (scale bar: 100 μm) and the mitochondrial morphology observed under transmission electron microscopy (TM) scale bar: 500 nm). (**B**–**E**) Quantitative RT-PCR of collagen III (**B**), Kim-1 (**C**), TNF-α (**D**), and IL-6 (**E**) mRNA normalized to expression of 18S, in the renal cortex (n=6). All data represent the mean ± standard deviation (SD). *p<0.01 vs. other groups. ZLRs; Zucker lean rats, ZDFRs; Zucker diabetic fatty rats.

Compared to the control ZLRs group that received saline, the ZDFRs saline group exhibited renal fibrosis in the tubulointerstitial area observed by Masson’s trichrome (MT) staining, and increased protein levels of anti-kidney injury molecule-1 (Kim-1) evaluated by immunohistochemistry (IHC) (Figure 1A). The ZDFRs saline group also had an altered mitochondrial morphology, such as mitochondrial swelling, in the proximal tubular cells (Figure 1A). In addition, the ZDFRs saline group exhibited an increased gene expression in the renal cortex of collagen III and Kim-1 (Figure 1B, 1C), and of the pro-inflammatory cytokines tumor necrosis factor-α (TNF-α) and interleukin-6 (IL-6) compared to the ZLRs saline group (Figure 1D, 1E). Importantly, administration of apigenin ameliorated these alterations in the ZDFRs group (Figure 1). In contrast, apigenin had no effect on renal fibrosis and Kim-1 expression in the tubulointerstitial area in the ZLRs group; a representative photograph of MT staining and Kim-1 IHC of ZLRs treated with apigenin is shown in Supplementary Figure 1.

### Apigenin downregulates CD38 in diabetic rats

**Figure 2:**
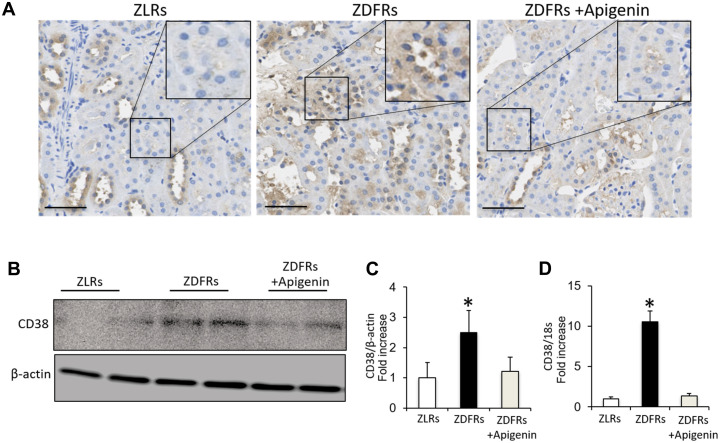
**Apigenin downregulates CD38 in diabetic rats.** (**A**) Representative photographs of immunohistochemical staining of CD38 in the tubulointerstitial area (scale bar: 100 μm). (**B**) Western blotting of CD38 and control β-actin in the renal cortex. (**C**) Densitometric evaluation of the western blotting shown in panel B (n=6). (**D**) Quantitative RT-PCR of CD38 mRNA normalized to 18S, in the renal cortex (n=6). All data represent the mean ± standard deviation (SD). *p<0.01 vs. other groups.

Compared to control ZLRs group that received saline, the kidneys of the ZDFRs saline group exhibited a significantly increased IHC of CD38 in tubular cells (Figure 2A), and upregulated CD38 gene and protein levels in the renal cortex (Figure 2B–2D). Administration of apigenin significantly reduced the CD38 expression in the kidneys of ZDFRs.

### Apigenin increases Sirt3 activity in diabetic rats

**Figure 3:**
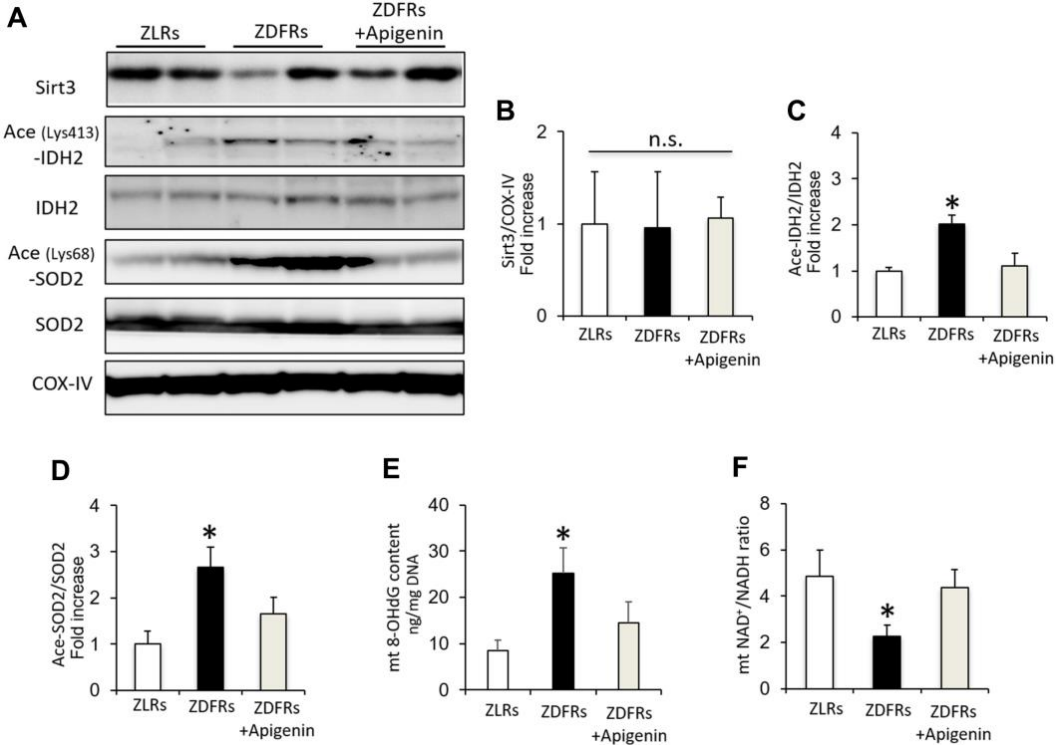
**Apigenin increases Sirt3 activity in diabetic rats.** (**A**) Western blots of Sirt3, acetylated (ace) (Lys413)-IDH2, IDH2, acetylated (ace) (Lys68)-SOD2, SOD2, and CoxIV from mitochondrial protein extracts obtained from rat renal cortex. (**B**–**D**) Densitometric evaluation of Sirt3 to CoxIV (**B**), Ace (Lys413)-IDH2 to IDH2 (**C**), and Ace (Lys68)-SOD2 to SOD2 (**D**) immunoblotting data shown in panel A (n=6). (**E**) 8-OHdG content in mitochondria (mt) adjusted to mtDNA in the renal cortex (n=6). (**F**) NAD^+^/NADH ratio in isolated mitochondrial protein extracts from rat renal cortex (n=6). All data represent the mean ± standard deviation (SD). *p<0.01 vs. other groups, n.s; not significant.

While the protein levels of Sirt3 in mitochondrial protein extracts from the renal cortex were similar among the groups (Figure 3A, 3B), the levels of acetylated IDH2 and SOD2 were significantly increased in the ZDFRs saline group compared to ZLRs saline group, indicating a reduced Sirt3 activity in diabetic rats (Figure 3A, 3C, 3D). The 8-OHdG content in mtDNA isolated from renal cortex was also significantly increased in ZDFRs administered saline compared to ZLRs treated with saline (Figure 3E). In contrast, the NAD^+^/NADH ratio was significantly decreased in mitochondrial protein extracts from renal cortex of the ZDFRs saline group compared to ZLRs saline group (Figure 3F). Importantly, apigenin suppressed the acetylated levels of IDH2 and SOD2, decreased the 8-OHdG content, and increased the NAD^+^/NADH ratio in ZDFRs (Figure 3), indicating that apigenin increases the NAD-dependent Sirt3 deacetylase activity in the kidneys of diabetic rats.

### Apigenin decreases CD38 expression, and increases Sirt3 activity and NAD^+^/NADH ratio in renal proximal tubular cells grown in high glucose

**Figure 4:**
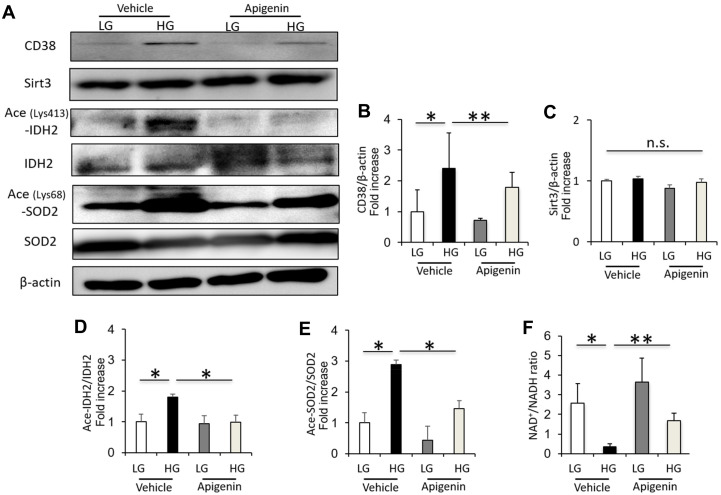
**Apigenin decreases CD38 expression, and increases Sirt3 activity and NAD^+^/NADH ratio in renal proximal tubular cells grown in high glucose.** (**A**) Western blots of CD38, Sirt3, ace (Lys413)-IDH2, IDH2, ace (Lys68)-SOD2, SOD2, and β-actin in HK-2 cells cultured in low glucose (LG; 5.55 mM) or high glucose (HG; 25 mM), with and without 10 μM apigenin. (**B**–**E**) Densitometric evaluation of CD38 to β-actin (**B**), Sirt3 to β-actin (**C**), Ace-IDH2 to IDH2 (**D**), and Ace-SOD2 to SOD2 (**E**) immunoblotting data shown in panel A (n=4). (**F**) Intracellular NAD^+^/NADH ratio in cultured HK-2 cells (n=4). All data represent the mean ± standard deviation (SD). *p<0.01 vs. the indicated group, **p<0.05 vs. the indicated group, n.s; not significant.

The CD38 expression and acetylated IDH2 and SOD2 levels were significantly increased in HK-2 cells cultured under high-glucose (HG) conditions compared to cells cultured in low-glucose (LG) (Figure 4A–4E). However, the Sirt3 expression in cultured HK-2 cells showed no change under LG and HG conditions (Figure 4A–4E). The intracellular NAD^+^/NADH ratio was significantly decreased in HK-2 cells cultured under HG conditions compared to cells cultured in LG (Figure 4F). Treatment with 10 μM apigenin significantly reduced the levels of CD38 and acetylated IDH2 and SOD2, and increased the intracellular NAD^+^/NADH ratio in HK-2 cells cultured under HG conditions (Figure 4), indicating that apigenin increases the Sirt3 deacetylase activity under HG conditions *in vitro*.

### CD38 knockdown increases Sirt3 activity and intracellular NAD^+^/NADH ratio in renal proximal tubular cells grown in high glucose

**Figure 5:**
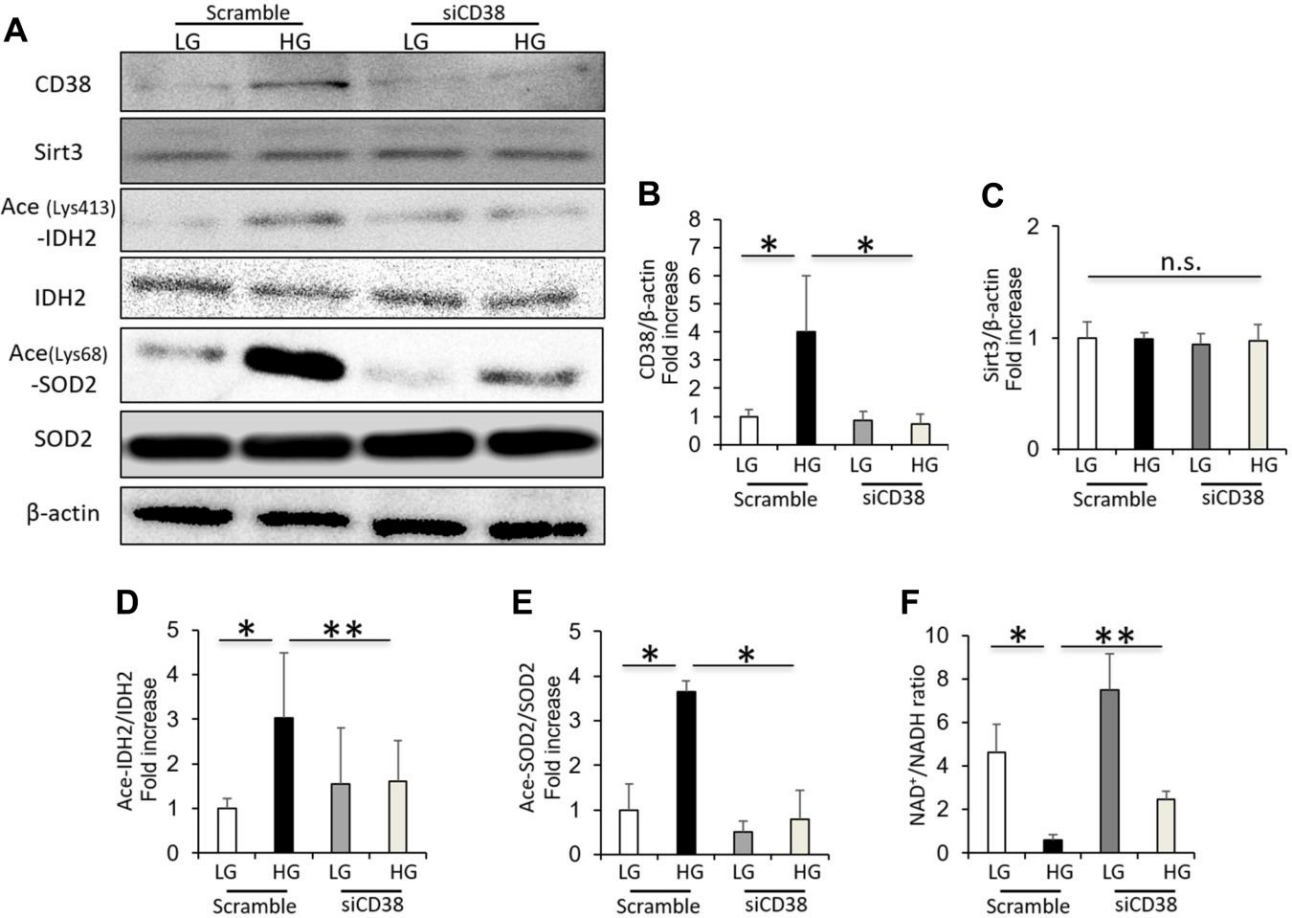
**CD38 knockdown increases Sirt3 activity and intracellular NAD^+^/NADH ratio in renal proximal tubular cells grown in high glucose.** (**A**) Western blots of CD38, Sirt3, ace (Lys413)-IDH2, IDH2, ace (Lys68)-SOD2, and β-actin in HK-2 cells cultured in low and high glucose after transfection using scrambled or CD38 siRNA. (**B**–**E**) Densitometric evaluation of CD38 to β-actin (**B**), Sirt3 to β-actin (**C**), Ace-IDH2 to IDH2 (**D**), and Ace-SOD2 to SOD2 (**E**) immunoblotting data shown in panel A (n=4). (**F**) Intracellular NAD^+^/NADH ratio in cultured HK-2 cells under the above conditions (n= 4). All data represent the mean ± standard deviation (SD). *p<0.01 vs. the indicated group, **p<0.05 vs. the indicated group, n.s; not significant.

The high glucose-induced CD38 expression in cultured HK-2 cells was suppressed by CD38 siRNA (Figure 5A, 5B). The Sirt3 expression in HK-2 cells was not dependent on the CD38 knockdown or glucose levels (Figure 5A, 5C). However, CD38 suppression by siRNA decreased the levels of acetylated IDH2 and SOD2 induced by HG in HK-2 cells (Figure 5A, 5D, 5E). In addition, CD38 suppression significantly increased the intracellular NAD^+^/NADH ratio in HK-2 cells cultured under HG conditions (Figure 5F). These data indicate that CD38 suppression increases the Sirt3 deacetylase activity and intracellular NAD^+^/NADH ratio in renal proximal tubular cells grown in high glucose conditions.

## DISCUSSION

Mitochondrial oxidative stress is closely associated with the pathogenesis of DKD. We have previously reported that mitochondrial oxidative stress is induced by decreased SOD2 and IDH2 activities associated with the decreased intracellular NAD^+^/NADH ratio and Sirt3 activity in the kidneys of diabetic rats [9]. The mitochondrial oxidative stress in diabetic kidneys is accompanied by increased levels of the NAD^+^-degrading enzyme CD38, in both glomerular and tubular cells [9]. In this study, we have focused on renal tubular cells, because tubular cell damage, rather than glomerular damage, is associated with the progression of DKD and renal function decline [5, 18]. We have found that the increased CD38 expression in renal tubular cells plays a crucial role in the pathogenesis of DKD caused by mitochondrial oxidative stress, which is associated with a reduction in the NAD^+^/NADH ratio and Sirt3 activation.

CD38 is one of the main NAD^+^-degrading enzymes in mammalian tissues, including liver, brain, heart and kidney [12, 19]. CD38 is localized in cell membrane, nucleus, and mitochondria [12, 20, 21], and plays a key role in several physiological processes, including immune responses, inflammation, cancer, and metabolic disease [11, 22–24]. Additionally, a previous study has shown that CD38 levels are increased in mouse tissues, including liver, adipose tissues, and skeletal muscles during aging, and suggested that CD38 is involved in the age-related NAD^+^ decline by using CD38 knockout mice [13]. Moreover, the increased levels of CD38 in aging mice correlated with the development of mitochondrial dysfunction, which partially occurred through a reduction in Sirt3 activity, resulting in age-related metabolic derangement [13]. Another report demonstrated that CD38 inhibition by apigenin increased the intracellular NAD^+^ levels and improved several aspects of glucose and lipid homeostasis in high-fat diet-induced obese mice [17]. We have previously shown for the first time that the CD38 expression is increased in the kidneys of the ZDFRs diabetic rats [9]. In this study, we have found that the increased expression of CD38 is associated with a decreased Sirt3 deacetylase activity, as indicated by the acetylation levels of the mitochondrial anti-oxidative enzymes IDH2 and SOD2 in diabetic kidneys. Administration of apigenin reduced the CD38 expression and mitochondrial oxidative stress in diabetic kidneys, resulting in improved diabetes-induced renal injuries, such as tubulointerstitial fibrosis, tubular damage, inflammation, and urinary albumin/L-FABP excretion. Since the inhibitory effect of apigenin on HbA1c levels was only modest, it seems likely that apigenin exerted the beneficial effect on diabetic renal injury independently of its effect on glucose levels.

What is the mechanism by which diabetes and high glucose lead to CD38 overexpression in kidneys and tubular cells? Previous reports have shown that lipopolysaccharides (LPS) and inflammatory cytokines, such as TNF-α, are potent inducers of CD38 [23–26]. Since CD38 is highly expressed in inflammatory cells, it is possible that a low-grade inflammation may lead to an increased expression of CD38 in renal tubular cells. It has been proposed that there is an increase in the levels of inflammatory cytokines and chemokines in diabetic kidneys [27, 28]. Our results show that the expression of inflammatory genes TNF-α and IL-6 is increased in the renal cortex of diabetic rats. Therefore, one possibility is that inflammatory cytokines, including TNF-α, may be the drivers of the increased CD38 expression in diabetic renal tubular cells. Moreover, the inhibition of CD38 expression in diabetic kidneys by apigenin may be mediated by a reduced renal inflammation. Flavonoids, including apigenin, can directly scavenge ROS [29, 30]. Previous studies have demonstrated that apigenin protects renal tubular cells against HG-induced oxidative stress via regulation of the NF-E2-related factor 2 pathway [31]. Therefore, apigenin might improve mitochondrial oxidative stress independently of its effects on CD38 inhibition.

In conclusion, our results provide new evidence that CD38 plays a crucial role in the pathogenesis of DKD caused by mitochondrial oxidative stress by inhibiting Sirt3 activity in renal tubular cells. Apigenin ameliorates the diabetes-induced renal tubular injury by reducing mitochondrial oxidative stress via CD38-mediated Sirt3 activation. Thus, CD38 inhibition may serve as a possible therapeutic strategy for the treatment of DKD. However, apigenin is not a specific inhibitor of CD38. A recent report has demonstrated that in mice, the CD38-specific inhibitor 78c improves several physiological and metabolic parameters linked to aging, including glucose tolerance, muscle function, exercise capacity, and cardiac function [32]. Therefore, further studies using specific inhibition of CD38, or CD38 knockout animals, are necessary to elucidate the role of CD38 in the development of DKD.

## MATERIALS AND METHODS

### Antibodies and reagents

Cytochrome C oxidase subunit IV (CoxIV), β-actin and IDH2 antibodies, and acetylated lysine antibodies were purchased from Cell Signaling Technology (Beverly, MA, USA). Anti-acetylated SOD2 (Lys-68) antibody was obtained from Abcam (Cambridge, MA, USA). The antibodies against Sirt3 and CD38 (M-19) used for western blotting and the antibody against CD38 (H-11) used for immunohistochemistry were purchased from Santa Cruz Biotechnology (Santa Cruz, CA, USA). The anti-acetylated IDH2 (Lys-413) antibody was obtained from GeneTel Laboratories LLC (Madison, WI, USA). The anti-SOD2 antibody was purchased from Enzo Life Science (New York, NY, USA). The anti-Kim-1 antibody was obtained from R & D Systems, Inc. (Minneapolis, MN, USA). Apigenin was purchased from Enzo Life Science (New York, NY, USA).

### Animal experiments

Male Zucker Lean (*fa/+*) Rats (ZLRs) and male Zucker Diabetic Fatty (*fa/fa*) Rats (ZDFRs) were provided by the Ninox Pharmaceutical Company Biological Institute (Osaka, Japan) [9]. The animal studies were approved by the Research Center for Animal Life Science of Kanazawa Medical University. At 24 weeks of age, the ZDFRs and ZLRs rats were randomly divided into groups that received 20 mg/kg apigenin or control saline solution via oral gavage 5 days a week [33]. After 4 weeks of the administration of apigenin or saline, individual rats were placed in metabolic cages for urine collection. Thereafter, the rats were anesthetized with isoflurane, blood samples were collected, and the kidneys were dissected as reported previously [9].

### Biochemical measurements

HbA1c levels were measured using a DCA 2000 Analyzer (Siemens Medical Solutions Diagnostics, Tokyo, Japan) at the end of the experiment [27]. Urinary albumin, L-FABP, and plasma cystatin C levels were measured using ELISA kits (urinary albumin: NEPHRAT II; L-FABP: Exocell, Inc., Philadelphia, PA, USA; L- FABP: R & D Systems, Inc., Minneapolis, MN, USA; Cystatin C: Rat Cystatin C kit, Abcam, Cambridge, MA, USA) [27]. Urinary 8-OHdG concentration was measured by ELISA (8-OHdG Check, Institute for the Control of Aging, Shizuoka, Japan) [27]. Urinary Cr was measured by a Creatinine Companion kit (Exocell, Inc., Philadelphia, PA, USA).

### Morphological analysis, IHC, and transmission electron microscopy

Paraffin sections (3 μm-thick) of the kidney were stained with MT stain, and IHC was performed using antibodies against Kim-1 (1:100) and CD38 (1:100) [9]. The mitochondrial morphology in the proximal tubular cells was observed using transmission electron microscopy [9, 27].

### Mitochondria extraction and 8-OHdG analysis

Isolation of mitochondria from the renal cortex was performed using mitochondria extraction kits (Thermo Fisher Scientific, Rockford, IL, USA) [9]. Mitochondrial DNA (mtDNA) was extracted from the renal cortex using the mtDNA Extractor CT kit (Wako Pure Chemical Industries, Osaka, Japan) [9]. 8-OHdG levels in DNase I-digested mtDNA were determined by ELISA (High-Sensitive 8-OHdG Check, Institute for the Control of Aging, Shizuoka, Japan) [9].

### Western blot analysis and real-time PCR

Western blotting was performed using antibodies against CD38 (1:1000), β-actin (1:1000), Acetylated (Lys-413)-IDH2 (1:1000), IDH2 (1:1000), Acetylated (Lys-68)-SOD2 (1:1000), SOD2 (1:1000), Sirt3 (1:1000) and COX-IV (1:1000) as previously described [9]. Total RNA was isolated from the renal cortex, and cDNA synthesis and quantitative real-time PCR were performed. TaqMan probes for collagen III, CD38, Kim-1, IL-6 and TNF-α were purchased from Thermo Fisher Scientific (Waltham, MA, USA). The data were normalized to the level of 18S mRNA, which was used as an internal control, as previously described [9].

### NAD^+^/NADH ratio assay

NAD^+^ and NADH levels were measured using NAD^+^/NADH assay kits according to the manufacturer’s instructions (BioChain, Hayward, CA, USA) [9]. The principle of the method is based on a glucose dehydrogenase cycling reaction, in which tetrazolium dye 3-(4,5-dimethyl-2-thiazolyl)-2,5-diphenyltetrazolium bromide (MTT) is reduced by NADH in the presence of phenazine methosulfate. The product absorbance, measured at 565 nm, is proportionate to the NAD^+^ concentration of the sample [9].

### Cell culture

Human kidney proximal tubular HK-2 cells were obtained from the American Type Culture Collection (Manassas, VA, USA). HK-2 cells were maintained in Dulbecco’s modified Eagle medium (DMEM) containing 10% fetal bovine serum and 5.55 mM glucose [9]. Sub-confluent HK-2 cells in 35-cm culture dishes were exposed to serum-free DMEM containing 5.55 or 25 mM glucose for 48 hours, and cells were then treated with 10 μM apigenin or DMSO for 24 hours.

### Transfection with siRNA

HK-2 cells were seeded in six-well plates and incubated for 24 hours. Cells were transfected with siRNA against CD38 (siRNA human CD38, s119605; Thermo Fisher Scientific, Waltham, MA, USA) or control scrambled siRNA (Negative Control #1 siRNA: Ambion Inc., Austin, TX, USA) at a concentration of 100 nM using Lipofectamine 3000 (Invitrogen, Carlsbad, CA, USA) according to the manufacturer’s instructions [9]. After transfection, cells were incubated in DMEM containing 5.55 or 25 mM glucose for 48 hours.

### Statistical analysis

The data are expressed as the mean ± standard deviation (SD). One-way ANOVA followed by Tukey’s multiple comparison test was used to determine the significance of differences among three or more groups; p<0.05 was considered significant.

## Supplementary Material

Supplementary Figure 1
