# Supplementary material for: CD38 inhibition by apigenin ameliorates mitochondrial oxidative stress through restoration of the intracellular NAD+/NADH ratio and Sirt3 activity in renal tubular cells in diabetic rats
Source: Aging (Albany NY). 2020 Jun 7;12(12):11325–36. doi: 10.18632/aging.103410 (PMC7343471; doi:10.18632/aging.103410)
Supplement: Supplementary Figure 1 [file aging-12-103410-s001..pdf]

SUPPLEMENTARY FIGURE

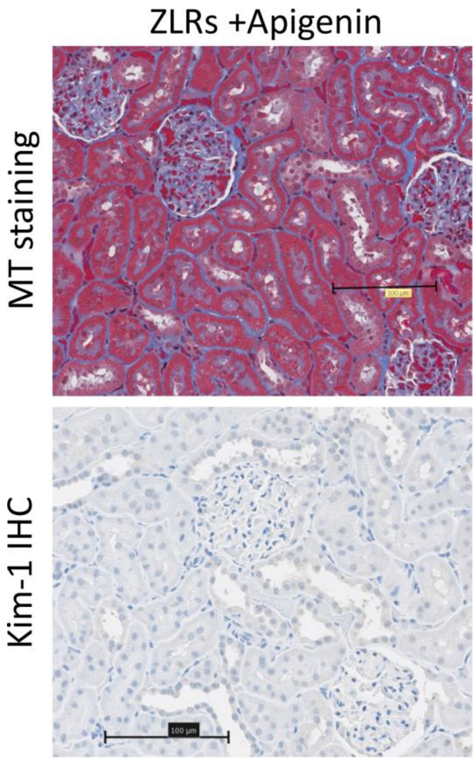

Supplementary Figure 1. Representative photograph of Masson's trichrome (MT) staining and kidney injury molecule-1 (Kim-1) immunohistochemistry (scale bar: 100 µm) in ZLRs treated with apigenin. ZLRs; Zucker lean rats.
